# Clinical characteristics and risk factors analysis of allergic bronchopulmonary aspergillosis combined with bronchiectasis

**DOI:** 10.3389/fmed.2025.1557241

**Published:** 2025-05-23

**Authors:** Qian He, Min Li, Jiaqi Cao, Ming Zhang, Chunlai Feng

**Affiliations:** ^1^Department of Respiratory and Critical Care Medicine, Third Affiliated Hospital of Soochow University, Changzhou, China; ^2^Department of Respiratory and Critical Care Medicine, Xishan People’s Hospital of Wuxi City, Wuxi, China

**Keywords:** allergic bronchopulmonary aspergillosis, bronchiectasis, risk factor, clinical characteristics, exacerbation

## Abstract

**Background:**

Allergic bronchopulmonary aspergillosis (ABPA) is a complex pulmonary disorder caused by a hypersensitivity reaction to *Aspergillus* colonizing the airways. Research on the clinical characteristics and risk factors of ABPA in patients with bronchiectasis is limited. This study aimed to investigate the clinical features and risk factors of ABPA in patients with bronchiectasis to improve clinical recognition.

**Methods:**

We retrospectively collected clinical data from bronchiectasis patients hospitalized at the Third Affiliated Hospital of Soochow University between September 2017 and December 2021.

**Results:**

A total of 251 patients were included in the analysis, of which 46 were confirmed to have ABPA with bronchiectasis. The remaining 205 patients served as control group. There were no significant differences in clinical symptoms (fever, cough, hemoptysis, chest pain, wheezing) between the two groups. However, blood eosinophil count and total IgE levels were significantly higher in the ABPA group compared to the control group. Both univariate and multivariable analyses revealed that a higher bronchiectasis severity index (BIS), frequent pet contact, hypoproteinemia, and *Aspergillus* colonization significantly increased the risk of developing ABPA in bronchiectasis.

**Conclusion:**

The clinical symptoms of ABPA in patients with bronchiectasis are clinically indistinguishable from those of non-ABPA bronchiectasis A higher BIS, frequent pet contact, hypoproteinemia, and *Aspergillus* colonization are identified as key risk factors for ABPA development in bronchiectasis.

## Introduction

*Aspergillus spp*. is ubiquitous in the environment and can release a significant numbers of spores into the air. Depending on the patient’s immune status, the inhalation of *Aspergillus* spores can lead to a variety of pulmonary disorders (1). Allergic bronchopulmonary aspergillosis (ABPA) is a Th2 cell-mediated hypersensitive immune response to *Aspergillus* colonization (2). Previous studies estimate the global burden of ABPA to be around 4.8 million people (3). Based on radiologic features, ABPA is generally classified into two types: serologic ABPA (ABPA-S) and ABPA with bronchiectasis (ABPA-B) (4). The most extensive study on in China to date indicates that the proportion of patients with ABPA-B (80.9%) significantly exceeds that of ABPA-S (19.1%) (5).

Bronchiectasis is a chronic lung disease characterized by permanent, irreversible bronchial dilatation, mucociliary dysfunction, and recurrent infections. As a result, patients with bronchiectasis are particularly vulnerable to infections, including bacterial and tuberculous mycobacterium infections (6). While bronchiectasis is often considered a structural complication of chronic airway inflammation, in ABPA, bronchiectasis predominantly arises from persistent mucoid impaction and recurrent immune-mediated damage triggered by *Aspergillus* colonization. This process leads to irreversible bronchial wall remodeling and dilatation over time. Studies suggest that the structural changes in ABPA-associated bronchiectasis are secondary to fungal-driven hypersensitivity rather than a pre-existing condition. For instance, The Cohort of Asian and Matched European Bronchiectasis (CAMEB) study highlighted that fungal colonization, particularly by *Aspergillus*, plays a critical role in both the pathogenesis and progression of bronchiectasis in ABPA (7). Consequently, the European guidelines recommend early screening for ABPA in bronchiectasis patients to mitigate irreversible lung damage caused by delayed diagnosis (8).

Therefore, it is essential to recognize the clinical characteristics of ABPA and identify its risk factors for ABPA in patients with bronchiectasis in order to develop effective strategies for monitoring and prevention. In this study, we aimed to analyze the clinical presentation, laboratory findings, and severity assessments while identifying the risk factors for ABPA in patients with bronchiectasis.

## Materials and methods

### Patients

This was a single-center, retrospective, observational study conducted at the Third Affiliated Hospital of Soochow University (Changzhou, China). We retrospectively collected clinical data from adult patients with bronchiectasis hospitalized between September 2017 and December 2021. The study was approved by the Institutional Review Board of Changzhou First People’s Hospital (No. 2021-009). Each patients signed an informed consent at the initiation of diagnosis, allowing for further clinical research using the clinical records.

Inclusion criteria: All patients were admitted due to the diagnosis or need for treatment of bronchiectasis, including exacerbation of cough, significant increase in sputum volume (purulent sputum), worsening of dyspnea, fever (body temperature > 38°C), etc. Bronchiectasis was diagnosed based on high-resolution computed tomography (HRCT) findings, defined as irreversible bronchial dilatation meeting at least one of the following criteria: (1). Bronchoarterial ratio > 1 (internal diameter of the bronchus exceeding the adjacent pulmonary artery). (2). Lack of bronchial tapering within 2 cm of the pleural surface. (3). Airway visibility within 1 cm of costal pleural surface or touching the mediastinal pleura (9). All computed tomography (CT) scans were independently reviewed by two experienced radiologists, with discrepancies resolved by consensus. Based on the modified International Society for Human and Animal Mycology (ISHAM) 2013 criteria proposed in 2016 (10). The inclusion criteria for ABPA with bronchiectasis were as follows: (1). A diagnosis of bronchiectasis; (2). Both of the following two additional criteria: elevated IgE levels against *Aspergillus fumigatus* (> 0.35 kUA/L) or positive *Aspergillus* skin prick test; total IgE levels > 1,000 IU/ml (or < 1,000 IU/ml if all other criteria are met); (3). At least two of the following three additional criteria: Presence of precipitating or IgG antibodies against *A. fumigatus* in serum; thoracic radiographic opacities consistent with ABPA (transient or permanent); or total eosinophil count > 0.5 × 10^9/L in oral steroid-naïve patients. The non-ABPA bronchiectasis patients present with bronchiectasis or concomitant infection on CT scans but without relevant evidence of *Aspergillus* infection.

Clinical data were collected for all patients, including demographic data, clinical symptoms, laboratory examinations, Pulmonary function, bronchiectasis severity index (BIS), chest CT findings, and treatment regimens. Bronchiectasis is classified as central if it is confined to the medial two-thirds or medial half of the lung. Frequent pet contact was defined as having contact with animals or pets more than three times per week over the past 12 months. Pets include in this definition are dogs, cats, rabbits, birds, cows, sheep, horses, and rodents.

### Statistical analysis

Continuous variables were expressed as the mean standard deviation. Categorical variables were expressed as proportions. The chi-squared test or Fisher s exact test was used to compare groups for count data and categorical variables. For continuous variables, the Student s *t*-test or the Mann Whitney U-test were used to compare groups, depending on whether the data were normally distributed. We performed Logistic regression models to analyze the risk factors associated with ABPA. All data were analyzed using SPSS version 23.0 (IBM Corp, Armonk, NY, United States). A *P*-value < 0.05 was considered to indicate statistical significance.

## Results

### Patient characteristics

A total of 251 patients were included in the study, with 46 diagnosed with ABPA and bronchiectasis, and 205 bronchiectasis patients without ABPA serving as controls. No significant differences were observed between the case and control groups in terms of sex, age, BMI, Extra-pulmonary comorbidities, and smoking history (Table 1). Compared with the control group, ABPA patients exhibited a significantly higher prevalence of pulmonary comorbidities (76.1% vs. 21.0%, *P* < 0.001), including asthma (54.3% vs. 10.7%) and prior pulmonary tuberculosis (21.7% vs. 10.2%). The proportion of patients using two or more antibiotics for more than 14 days within 6 months prior to diagnosis was significantly higher in the ABPA group compared to the control group (*p* < 0.001). Additionally, the BIS score was significantly higher in the ABPA group than in the control group. A history of frequent pet contact was reported by 50% of ABPA patients, which was significantly higher than in the control group (*p* < 0.001) (Table 1).

**TABLE 1:** Baseline characteristics of the study population.

Variables	ABPA group (*N* = 46)	Control group (*N* = 205)	*P*-value
Male	23 (50%)	105 (51.2)	0.88
Age (in years)	58.97 ± 10.15	60.68 ± 11.12	0.34
BMI (body mass index)	22.00 ± 3.60	21.59 ± 3.66	0.49
Pulmonary comorbidities	35 (76.1%)	43 (21.0%)	0.001
Asthma	25 (54.4%)	22 (10.7%)	–
Previous pulmonary tuberculosis	10 (21.7%)	21 (10.2%)	–
Extra-pulmonary comorbidities	13 (28.2%)	86 (42%)	0.09
Cardiovascular diseases	6 (13%)	43 (21%)	
Diabetes mellitus	4 (8.7%)	28 (13.7%)	–
Extrapulmonary malignant tumor	3 (6.5%)	15 (7.3%)	–
Using two or more antibiotics for more than 14 days within 6 months (before diagnosed)	28 (60.9%)	65 (31.7%)	< 0.001
Bronchiectasis severity index (BIS)	10 (5, 13)	6 (4, 10)	0.004
History of smoking	31 (67.4%)	156 (76.1%)	0.22
Frequent pet contact	23 (50%)	38 (18.5%)	< 0.001
Hypoproteinemia	27 (58.7%)	24 (11.7%)	< 0.001

### Clinical characteristics, laboratory and radiological findings

No significant differences in clinical symptoms (e.g., fever, cough, hemoptysis, chest pain, wheezing) were observed between the two groups. The most common clinical symptom was cough, which was present in 93.5% of the ABPA group compared to 90.7% in the control group.

**FIGURE 1:**
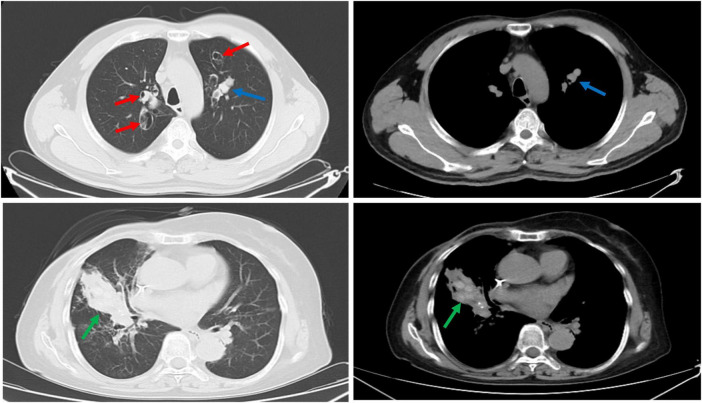
Schematic computed tomography (CT) of allergic bronchopulmonary aspergillosis (ABPA) with bronchiectasis: central bronchiectasis (red arrows); mucoid impaction (blue arrows) and high attenuation mucus (green arrows).

The blood eosinophils count and total IgE levels were significantly higher in the ABPA group compared to the control group (*P* < 0.001). All ABPA patients had elevated *Aspergillus*-specific IgE levels, whereas only 4.9% of bronchiectasis patients were positive. No significant differences were observed between the case and control groups in terms of blood white cell count, neutrophil count, C-reactive protein (CRP), and procalcitonin (PCT). Similarly, no differences in lung function (FEV1) were found between the two groups (Table 2). However, central bronchiectasis and high attenuation mucus were more frequent in the ABPA group (*P* < 0.001) (Figure 1).

**TABLE 2:** Clinical, laboratory examination, and radiographic differences between the two group.

Variables	ABPA group (*N* = 46)	Control group (*N* = 205)	*P*-value
**Clinical symptoms**			
Fever	5 (10.9%)	18 (8.8%)	0.58
Cough	43 (93.5%)	186 (90.7%)	0.77
Hemoptysis	21 (45.7%)	79 (38.5%)	0.37
Chest pain	3 (6.5%)	12 (5.9%)	0.74
Wheezing	19 (41.3%)	68 (33.2%)	0.30
**Laboratory examination**			
White cell count (× 10^9^/L)	7.55 ± 2.77	7.04 ± 2.61	0.24
Neutrophil count (× 10^9^/L)	5.25 ± 2.72	4.72 ± 2.53	0.21
Eosinophil count (× 10^9^/L)	0.90 ± 0.54	0.19 ± 0.41	< 0.001
C-reactive protein (CRP) (mg/L)	23.90 ± 45.72	30.78 ± 51.86	0.41
Procalcitonin (PCT) (ng/mL)	0.20 ± 0.31	0.15 ± 0.25	0.25
Total IgE levels (IU/mL)	1602.20 ± 933.64	84.59 ± 119.66	< 0.001
*Aspergillus*-specific IgE levels > 0.35 KU/L	46 (100%)	10 (4.9%)	< 0.001
*FEV1% predicted			
> 80%	11 (23.9%)	84 (41%)	0.15
50%–80%	21 (45.7%)	75 (36.5%)	–
30%–49%	12 (26.1%)	35 (17.1%)	–
< 30%	2 (4.3%)	11 (5.4%)	–
**Radiographic finding**			
Distribution of bronchiectasis			
Solitary (single lung)	9 (19.6%)	63 (30.7%)	0.11
Multiple (single lung)	12 (26.1%)	64 (31.3%)	–
Multiple (bilateral lung)	25 (54.3%)	78 (38%)	–
Central Bronchiectasis	41 (89.1%)	78 (38%)	< 0.001
Mucoid impaction	17 (37%)	20 (9.8%)	< 0.001
High attenuation mucus	15 (32.6%)	0	< 0.001
**Sputum culture**			
*Aspergillus spp*	5 (10.9%)	4 (2%)	0.01
*P. aeruginosa*	14 (30.4%)	35 (17.1%)	0.04

*FEV1, forced expiratory volume in 1 s.

### Univariate and multivariate logistic regression analysis of risk factors for ABPA with bronchiectasis

Table 3 summarizes the risk factors for ABPA in bronchiectasis. Both univariate and multivariable analyses identified factors significantly associated with ABPA comorbidity in patients with bronchiectasis, including higher BIS, frequent pet contact, hypoproteinemia, and *Aspergillus* colonization.

**TABLE 3:** Univariate and multivariate analysis of risk factors for allergic bronchopulmonary aspergillosis (ABPA) with bronchiectasis.

Univariate analysis			Multivariate analysis	
**Risk factor**	**OR (95% CI)**	***P*-value**	**OR (95% CI)**	***P*-value**
Gender	1.01 (0.53,1.91)	0.98	–	–
Age	0.99 (0.96,1.02)	0.34	–	–
BMI	1.03 (0.95,1.13)	0.49	–	–
BIS	1.14 (1.05,1.23)	0.002	9.97 (2.72,36.48)	0.001
CRP	1.0 (0.99,1.00)	0.41	–	–
Pulmonary comorbidities	1.77 (0.93,3.38)	0.08	–	–
Frequent pet contact	4.39 (2.23,8.65)	< 0.001	4.82 (1.02,22.79)	0.04
Hypoproteinemia	6.21 (3.11,12.36)	< 0.001	4.81 (1.05,22.12)	0.04
*P. Aeruginosa* colonization	2.13 (1.03,4.39)	0.04	0.35 (0.03,4.14)	0.40
*Aspergillus* colonization	6.13 (1.58,23.81)	0.009	35.03 (1.70,72.32)	0.02

## Discussion

Allergic bronchopulmonary aspergillosis is an allergic lung disease caused by sensitization to *Aspergillus spp.* Antigens (11). A study has reported that more than half of ABPA cases in bronchiectasis patients are misdiagnosed due to atypical clinical symptoms (12). In this study, we analyzed the clinical data of ABPA patients. Multivariate analysis identified higher BIS, the use of two or more antibiotics for more than 14 days within 6 months, frequent pet contact and hypoproteinemia as risk factors for ABPA in patients with bronchiectasis.

Previous studies have demonstrated that patients with ABPA often have other pulmonary comorbidities, with asthma being the most common (11, 13). This aligns with our findings, which indicate that 54% of ABPA patients had a history of asthma. However, a Japanese study reported that 81% of ABPA patients with bronchiectasis had asthma (14). While in India, the prevalence of ABPA with asthma is as high as 93% (15). Several factors may explain this discrepancy: firstly, the relatively low diagnosis and treatment rates of asthma in certain areas of China (16), and secondly, the fact that nearly 22% of the patients in the ABPA group also had comorbid pulmonary tuberculosis, which has been highly prevalent in China in recent years. A study of the Chinese population indicated that 50%–60% of ABPA patients are misdiagnosed for four to 5 years (5), often leading to repeated antibiotic treatments. Our study also explored the relationship between frequent contact with pets and ABPA, showing that frequent pet contact was significantly associated with ABPA in bronchiectasis patients. This result is consistent with studies by Grehn and coworkers, who found that frequent pet contact was not linked to *Aspergillus* colonization, it was strongly associated with ABPA in cystic fibrosis patients (17). Previous study has identified *Aspergillus spp.* as the most common fungi found genus in dogs’ skin and hair (18). Additionally, our study highlights hypoproteinemia as a risk factor for ABPA development. A previous study on patients with hematological malignancies found that those who developed invasive pulmonary fungal infections had a significantly higher prevalence of comorbid hypoproteinemia compared to controls (70.8% vs. 21.7%) (19). Other studies suggest that hypoproteinemia is often closely associated with poor prognosis in pulmonary aspergillosis (20, 21). As is well-known, bronchiectasis patients with higher BIS often experience more severe conditions (22). Menendez and coworkers found that BIS is a risk factors for acute bronchiectasis exacerbation (23). A study on exacerbation of bronchiectasis due to influenza revealed that 55% of patients had co-infection, with 7% involving fungal infections (24). Our study similarly found a strong correlation between high BIS and the occurrence of ABPA in patients with bronchiectasis. ABPA is recognized as a serious complication of *Aspergillus* airway colonization (25, 26). In our study, we observed that *Aspergillus* colonization was more common in ABPA patients than in controls. *P. aeruginosa* and *A. fumigatus* frequently coexist and interact within the lungs (27). However, our multivariate analysis did not show a significant relationship between *P. aeruginosa* colonization and ABPA, a finding similar to Jubin’s study. Jubin and coworkers also found no correlation between ABPA and bronchial flora (including *Staphylococcus aureus; Pseudomonas aeruginosa; Stenotrophomonas maltophilia; Alcaligenes xylosoxydans; Candida species*) in patients with cystic fibrosis (28).

In ABPA, the interplay between fungal colonization and structural lung damage is complex. Chronic mucoid impaction and hypersensitivity-driven inflammation caused by *Aspergillus* colonization can lead to bronchial wall damage and subsequent bronchiectasis. Conversely, once bronchiectasis develops, the distorted airway architecture and impaired mucociliary clearance create a favorable environment for persistent fungal colonization, perpetuating a vicious cycle of inflammation and structural remodeling. Increased antigen release from colonized *Aspergillus* contributes to the destruction of airway epithelial cells and the activation of T lymphocytes, which triggers an inflammatory response in the airway and lung tissue, ultimately leading to bronchospasm (29). A key diagnostic criterion for ABPA in the most recent management guidelines is an eosinophil count exceeding 500 μL. In this research, the eosinophil count was significantly higher in the ABPA group (900 ± 540 μL) compared to the control group (190 ± 410 μL). However, 14 ABPA patients had eosinophil counts below 500 cells/μL, which was likely due to ongoing hormone therapy or being in a stable disease phase. Similarly, Zhang and coworkers reported that 34% of ABPA patients had eosinophil counts under 500 cells/μL (30). The International Society for Human and Animal Mycology suggests that a total serum IgE levels above 1,000 IU/mL is useful cutoff for diagnosing ABPA. In our findings, the mean total IgE level in ABPA patients was 1602.20 ± 933.64 IU/mL. Oguma reported a median IgE level of 1,913 IU/mL in Japanese ABPA patients (14). In contrast, a study of Korean patients found a median IgE level of 927 IU/mL (31), which may be attributed to the small study population (only 10 patients). In addition, 10 patients with ABPA in our study had total IgE below 1,000 IU/mL. Oguma’s study also found that nearly a third of ABPA cases IgE levels < 1,000 IU/mL (14), which could be related to a later-onset asthma in ABPA patients with bronchiectasis.

Given the similarity of clinical symptoms and imaging features of ABPA with bronchiectasis to other diseases, recognizing and diagnosing ABPA remains a challenge (10). Studies have reported that the most common misdiagnosed in China include bronchiectasis and pneumonia (5, 11). Therefore, analyzing imaging results is crucial for diagnosis, as clinicians who are unfamiliar with the imaging features of ABPA may misdiagnose the condition during the initial visit. Many studies have highlighted central bronchiectasis as a hallmark of ABPA (8, 32). However, some studies suggest that ABPA-associated bronchiectasis can expand to the periphery in 26%–39% of cases (10, 33), reducing its discriminatory value. Our study found that central bronchiectasis and high-attenuation mucus were more commonly observed in the ABPA group. Studies also emphasize that mucus plugs remain an important radiological marker for distinguish ABPA from other causes of bronchiectasis (34, 35). High-attenuation mucus (HAM), defined as mucus denser than paraspinal skeletal muscle, is associated with frequent exacerbations of ABPA (36). Excess mucus and distortion of central airway structure facilitate fungal colonization, leading to an inflammatory reaction.

However, this study has some limitations. First, it is a single-center study with a relatively small sample size of ABPA patients, which may reduce the statistical power. Secondly, reliance on electronic medical records data may lead to incomplete information (e.g., unrecorded occupational exposures or lifestyle habits of patients). Therefore, future research should focus on expanding the sample size through multi-center collaboration and adopting a prospective cohort design to include more factors.

## Conclusion

In summary, The clinical symptoms of ABPA in patients with bronchiectasis are clinically indistinguishable from those of non-ABPA bronchiectasis. The risk factors for incident ABPA in bronchiectasis include a higher BIS, frequent pet contact, hypoproteinemia and *Aspergillus* colonization.

## Data Availability

The raw data supporting the conclusions of this article will be made available by the authors, without undue reservation.
